# The current state and future direction of childcare for cancer patients: a narrative review

**DOI:** 10.1007/s00520-025-09174-6

**Published:** 2025-01-30

**Authors:** Hasiya Yusuf, Abhishek Kumar

**Affiliations:** Albert Einstein College of Medicine/Jacobi Medical Center, Building 1, 3N20, 1400 Pelham Parkway S, Bronx, NY USA

**Keywords:** Childcare, Cancer, Parents with cancer, Childcare services

## Abstract

**Purpose:**

One in four patients diagnosed with cancer are parents to dependent children. For these patients, childcare services are needed to overcome the time demands of cancer treatment. Despite the childcare support needs and its potential impact on treatment outcomes, targeted childcare services for cancer patients remain limited. This review highlights the state of childcare services and strategies to address the current chasm in childcare for parents diagnosed with cancer.

**Methods:**

A comprehensive search of PubMed, Google Scholar, and Embase was conducted and 77 studies in the English Language on Childcare services for parents with cancer published between January 1990 and May 2024 were identified and reviewed.

Findings.

The burden of cancer diagnosis and cancer treatment imposes physical, psychological, financial, and time constraints on cancer patients with young dependents. Many cancer patients with children miss treatment appointments and encounter treatment delays due to challenges with childcare. Limited access to childcare is further exacerbated by the financial and time toxicities of cancer and unconventional treatment needs such as emergency department visits, fatigue, and other complications of cancer treatment. So far, only one cancer-center-linked childcare program exists in the U.S., highlighting the scale of unmet need for childcare support in cancer patients.

**Conclusion:**

and relevance.

Providing non-traditional childcare services, home-based or hospital-based childcare structures, and financial assistance through medical institutions, professional organizations, insurance payers, and government-funded programs could bridge the current gap in childcare needs for parents with a cancer diagnosis.

## Introduction

The evolving epidemiological shift from communicable to non-communicable diseases (NCD) is reflected in the rising global incidence of cancers [1, 2]. More than 70% of annual recorded deaths worldwide are due to NCD, and heart disease and cancers account for the majority of these deaths [3]. Cancer is the second leading cause of death both globally and in the United States (U.S.) [3–6]. According to the World Health Organization’s (WHO) global report, 9.3 million people die annually from cancer [2]. Correspondingly, nearly two million cancers are diagnosed annually in the U.S., and available data indicate that 40% of the U.S. population will be diagnosed with cancer at some point in their lives [3–5].

Cancer diagnoses in persons younger than 50 years, also known as early-onset cancers, have been on the rise in the last three decades [7, 8]. Between 1990 and 2019, the incidence of early-onset cancers rose by 79.1% from 1.82 million to 3.26 million cases globally [8]. The U.S. had the highest age-adjusted incidence rates of early-onset cancers of any country in 2019, and this is projected to steadily increase until 2030 [8]. Consequently, more persons of reproductive age who are also likely to be parents to dependent children are living with cancer [7]. About 25% of cancer patients are parents to children under 18 years, and the majority of parents have two or more children under 10 years [9–11]. At this crucial developmental stage, children tend to be dependent on parents or other adults for emotional, physical, physiological, and social support [12, 13].

Parental cancer diagnosis has both physical and psychosocial effects on children. It has been linked to academic disruptions, childhood anxiety, depression, and emotional distress that may persist for up to 6 years after cancer diagnosis [10, 14]. Children of cancer patients are also at increased risk of losing a parent. In 2020 alone, an estimated 28,000 children in North America were orphaned from maternal cancer deaths [15]. Early parental death has been linked to lower educational attainment, lower rates of employment, and poor socioeconomic status [16].

Conversely, parenthood is a significant stressor for patients with cancer. Cancer patients who are primary caregivers to their children have a 97% increased risk of depression and are more likely to miss cancer treatment appointments [9, 11, 17]. Hence, cancer patients with dependent children simultaneously grapple with the challenges of childrearing, the effect of parental cancer on the children, the existential demands of a cancer diagnosis, and its potentially detrimental impact on cancer outcomes [9, 10].

Time toxicity in cancer describes the time spent undergoing cancer-related care, including chemotherapy, radiotherapy, routine follow-up visits, and other direct and indirect aspects of care [18, 19]. The time toxicity of cancer can be astronomical, reaching 20% of a patient’s entire lifetime for certain cancers, although highly variable and dependent on cancer stage and cancer-associated morbidity [18–20]. For parents of minor children, the time toxicity of cancer can have far-reaching consequences, causing disruptions to family functioning and limitations in fulfilling parental responsibilities [21]. Moreover, when faced with the difficult choice between parental and treatment obligations, parents, particularly women, often strive to prioritize their children’s needs [22]. The choice is likely motivated by the desire to minimize disruptions to family structure and mitigate the impact of a cancer diagnosis on the well-being of the children.

While understandable, hierarchizing childcare responsibilities leads to treatment delays, which negatively affect overall cancer treatment outcomes [11, 21]. As little as a 4-week delay in head and neck surgery and mastectomy is associated with a 6–8% increased risk of death [23]. Mortality risk is even higher for delays in systemic therapy of many cancers, reaching 56% for a 1-month delay in neoadjuvant breast cancer treatment [23]. Despite the profound implication of delayed or missed treatment, nearly 50% of cancer patients with children report rescheduling various oncology appointments due to a lack of childcare support [11]. Moreover, one in seven patients on radiotherapy treatment disclosed missing appointments for similar reasons [9]. Despite the gaping childcare support needs of this unique patient population and its potential consequences for cancer treatment outcomes, targeted childcare services for cancer patients are practically non-existent both in the U.S. and across North America [24–26]. This narrative review sought to highlight the state of childcare services for cancer patients with dependent children. It also highlights potential strategies for addressing the current chasm in childcare services for cancer-afflicted parents.

## Methods

We conducted a comprehensive search of studies on childcare for patients undergoing cancer treatment, published between January 1990 and May 2024, using PubMed, EMBASE, and Google Scholar databases. The search utilized keywords such as “childcare,” “cancer,” “parents with cancer,” “childcare services,” and “cancer patients with minor children.” The search was limited to articles published in English. Included publications comprised narrative, literature, and systematic reviews, as well as prospective and retrospective studies and randomized clinical trials. Additionally, a few relevant abstracts were also incorporated due to the scarcity of research on childcare in the context of cancer. A total of 77 articles met the inclusion criteria and were included in the narrative review. This study did not require ethical approval or informed consent.

### Demographics of cancer patients with minor children

Prevailing cultural and societal norms largely influence the distribution of caregiving burdens and responsibilities [27]. In many countries, women assume a disproportionate role in caregiving, including raising children [28, 29]. Even in the U.S., more than 90% of primary caregivers of children are women [28]. This caregiver demographic is reflected in cancer patients, where women make up a higher proportion of subjects in research on childrearing in cancer patients [30–35]. The few studies that recruit parents of both sexes also demonstrate a preponderance of women, representing more than 80% of subjects [9, 11]. Thus, mothers with cancer play a predominant role in caring for their children, and this role does not appear to diminish with a cancer diagnosis [32]. Predictably, most cancer patients with dependent children are in the reproductive age group, typically in the third and fourth decades of life [9, 11, 22, 35, 36]. The vast majority have breast cancer, although gynecological malignancies are also common [9, 11, 22, 37]. Indeed, one in every three patients with a diagnosis of breast cancer is a parent to dependent children [38].

Similar to the generality of cancer studies, most studies on childcare in cancer patients include patients in active treatment (curative or palliative) [37]. Employment or financial status is not routinely reported for parents with cancer. Still, the limited evidence demonstrates relatively low employment rates in this population, with one study reporting an employment rate of less than 30% [9]. While it is unclear if low unemployment rates in parents receiving cancer treatment are due to job losses related to cancer diagnoses, the link between active cancer treatment and resultant unemployment is well established [39, 40]. One in three patients becomes unemployed following a cancer diagnosis [41]. In a dyadic study to assess the needs of couples with a parent living with advanced cancer, employment rate was nearly 50% higher in the partners without cancer when compared to cancer patients independent of gender [42].

### State of childcare services and its impact on cancer patients

Quality childcare services remain one of the basic needs of families across the world. Lack of childcare services has been linked to higher unemployment rates, particularly for women who constitute the generality of primary caregivers [28, 29]. As a result, providing high-quality and affordable non-parental childcare became increasingly important in promoting women’s participation in the workforce [43, 44]. As childcare support services evolved, women’s participation in the workforce progressively increased over the past five decades [45]. Despite the remarkable progress, the issues of childcare availability, accessibility, and affordability are re-emerging [43].

**Fig. 1:**
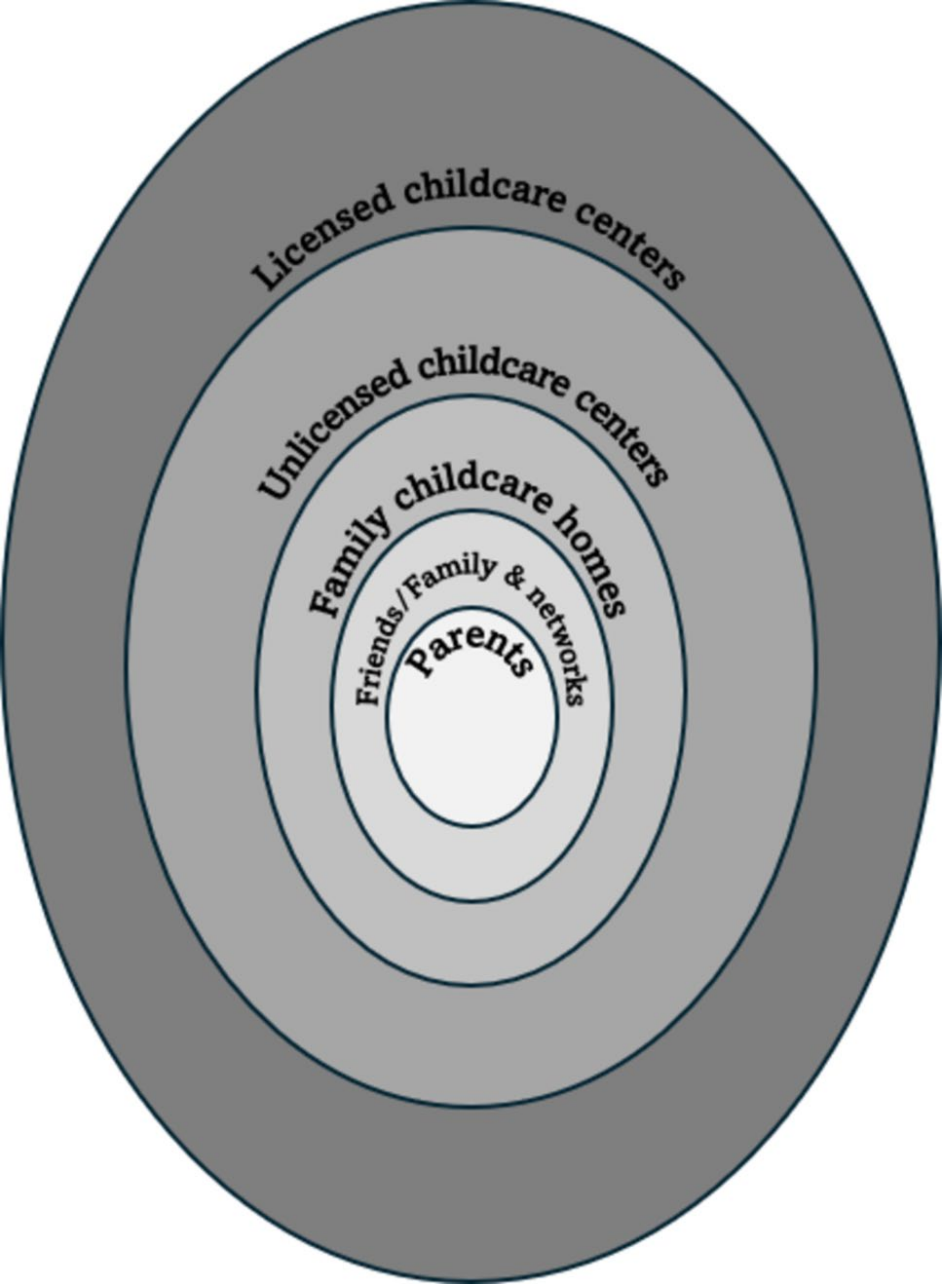
Levels of childcare services used by families in the U.S

Disparities in accessing childcare services exist across countries of the world, even in the most developed countries [46]. In Eastern Europe, no greater than 10% of children under 2 years are enrolled in formal childcare centers, a sharp contrast to countries like Denmark, Iceland, and Sweden, where more than 60% of children of similar ages have access to childcare services [46]. The U.S. sits somewhere in the middle, with 38% of children in the age group in formal childcare centers. In absolute numbers, approximately 11 million children under 5 are enrolled in some form of childcare program in the U.S. (Fig. 1 shows the different levels of childcare services available to families) [43]. However, most (47%) are not enrolled in professional center-based childcare systems but are cared for by relatives such as grandparents or non-working parents. Only 35% of children are enrolled in center-based care [43, 47].

**Fig. 2:**
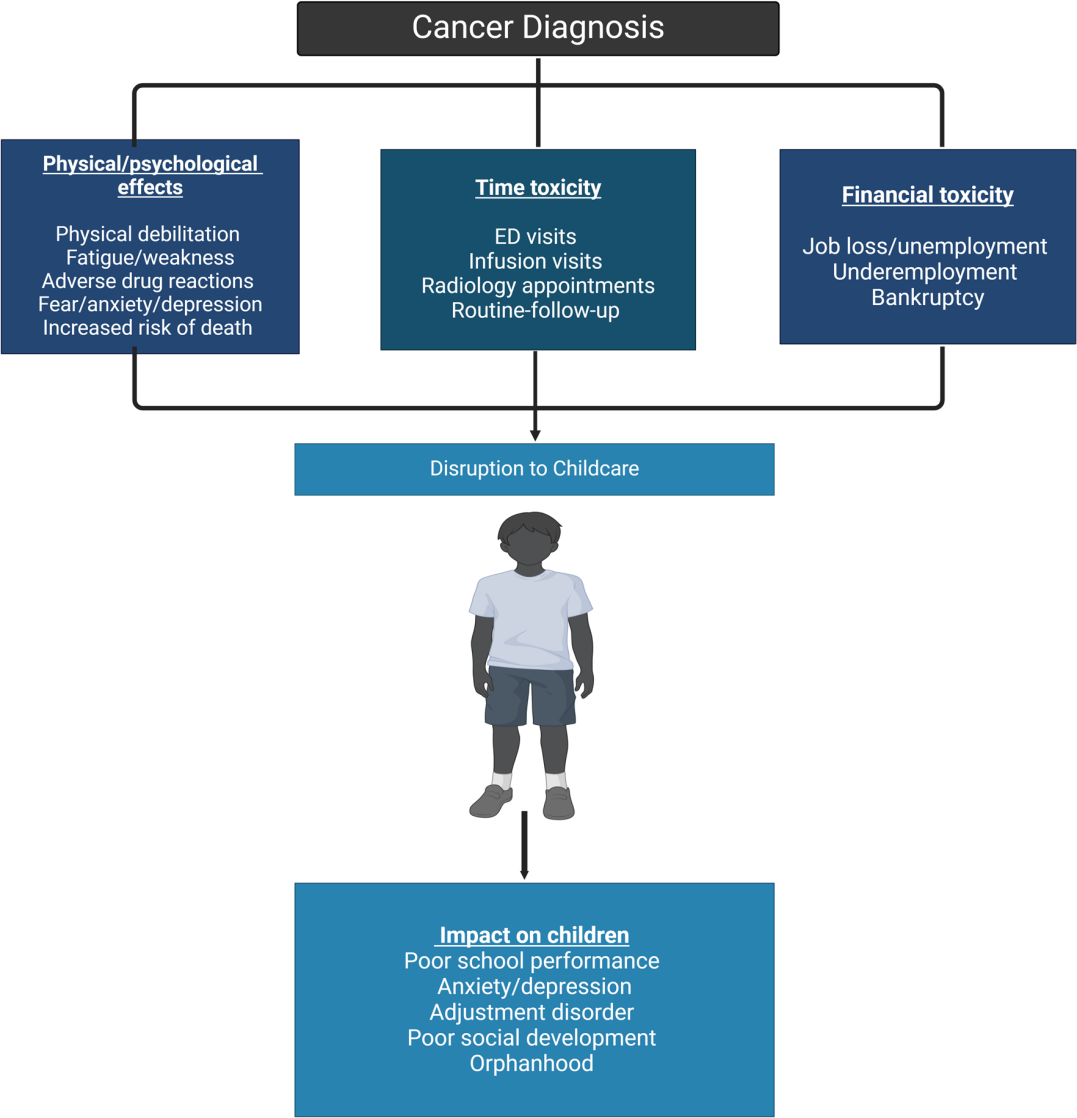
Impact of parental cancer diagnosis

Lack of access to childcare services limits employability and is associated with missing work [43, 44]. Conversely, unemployment leads to financial vulnerability and the inability to afford childcare, culminating in a vicious cycle [48, 49]. Cancer patients with young dependents epitomize this double blow of high risk of unemployment and the financial toxicity associated with a cancer diagnosis [39, 40]. Financial toxicity describes the economic burden of cancer treatment on cancer patients and their families [40, 50, 51]. Financial toxicity can be direct (cost of chemotherapy, radiation treatment, consultations, laboratory workup, and imaging costs) or indirect (underemployment or job loss from incapacitation) (see Fig. 2) [40]. Nearly 50% of cancer survivors endorse experiencing financial toxicity, which directly impacts childcare affordability [50]. The risk factors for financial toxicity in cancer patients (younger age, unemployment or low socio-economic status, and being a racial minority) overlap with the barriers to accessing childcare [50–52]. The combination of cancer-induced economic strain and the cost of childcare is a crushing blow to the cancer patient, serving as a driver of widening financial vulnerability and bankruptcy risk, which has also been linked to increased mortality [51, 53].

According to the U.S. Department of Health and Human Services (DHHS), a family should spend no more than 7% of their earnings on childcare to be considered affordable [54]. However, data show that childcare spending exceeds the recommended benchmark in 41 of 50 states [43, 47]. The U.S. Census Bureau reports that childcare costs can range between $5357 and $17,171 for a single child, reaching $20,000 or higher in Massachusetts and Washington D.C [55]. Estimates are considerably higher for single parents who spend more than 35% of household income on childcare costs and up to $30,0000 on childcare for a single child based on the child’s age and state of residence [43]. Up to 60% of the prohibitive cost of childcare is borne by parents [43, 48]. The cost of childcare is further exacerbated by the prolonged closure of childcare centers during the COVID-19 pandemic, a trend that persists post-pandemic [56]. Although data on childcare expenses specific to cancer patients are scarce, it can be estimated that childcare expenditures would be higher in this group compared to the general population.

It can also be argued that the cost or inaccessibility of childcare services has worse consequences for cancer patients compared to the general population due to the time toxicity of cancer [18, 20]. For example, cancer patients with minor children report multiple missed treatment appointments and rescheduling due to challenges with childcare [9, 34]. In addition, 20% of parents with children who require childcare work unconventional hours that are disconnected from the standard business hours of most childcare service providers [57]. Comparably, cancer patients often have unconventional or difficult-to-schedule treatment needs that place them in the pool of parents for which unconventional childcare is critical. Chemotherapy, radiotherapy, or follow-up appointments usually coincide with standard workday schedules, but a cancer diagnosis puts patients at added risk for unpredictable medical emergencies and treatment complications [58–62]. Fatigue from cancer treatment or cancer complications can constrain patients, contributing to the already tricky landscape of navigating childcare [21, 22].

In addition, childcare needs during cancer treatment vary based on family dynamics and the age or developmental stages of children [32]. Most parents depend on external support systems for childcare through mobilizing close social networks such as relatives, friends, neighbors, and colleagues [21, 63]. Family and friends account for more than 80% of childcare support systems for parents undergoing cancer treatment [63]. However, reliance on the generosity and goodwill of social networks is usually insufficient to meet the overwhelming childcare needs for various reasons. First, emotional support, rather than childcare support, is the predominant form of support received by parents undergoing cancer treatment from their closest support systems [63]. As a result, childcare needs remain largely unaddressed even in the presence of supportive social networks. While emotional support has its role in decreasing anxiety and has a positive impact on the overall outcome for cancer patients [63], ensuring the well-being and care of their children remains the utmost priority of parents with cancer, independent of cancer type or stage [37].

The degree of support received from family and close social networks is dependent on availability and individual and personal needs. For example, in a study to explore the nature of support provided by the social networks of parents with a cancer diagnosis, less than 50% of social networks offered social support, and childcare support was infrequent. Demographically, 90% of their support system had jobs of their own, and more than half had dependent children [63]. Hence, childcare support is hampered by the personal, family, and work demands of patients’ social networks. In addition, childcare support through close network systems does not appear to decrease the number of missed appointments and, therefore, may not be an optimal strategy for addressing the childcare needs of cancer patients [34].

### Hospital or institution-based childcare services

Many hospitals across the U.S. are bequeathed with childcare centers for employees. In comparison, patient-directed childcare support services are limited at best and, in many places, non-existent [24, 26]. In a survey of parent-accessible on-site childcare services across hospitals in North America, 77% of the 161 hospitals with childcare support services identified were pediatric hospitals [26]. Only one childcare center was associated with a cancer facility, highlighting the scale of unmet childcare needs of cancer patients with dependent children. A literature review to assess the state of childcare or childminding programs across designated cancer treatment centers in Canada reported similar trends: a predominance of pediatric hospital-associated childcare centers and a complete absence of childcare support structures across adult cancer treatment centers [64]. The near complete absence of childcare services for parents undergoing cancer treatment translates to a potentially considerable setback in cancer treatment for 25% of cancer patients. 

A dichotomy exists between providers’ perceptions and the reality of the impact of unmet childcare needs on treatment adherence and outcomes. In a survey of radiation oncology departments of cancer centers across the U.S., more than 90% of centers reported offering childcare referrals to cancer patients, and one-third of centers reported providing financial assistance for childcare to patients [24]. However, it is worth noting that 55% of the same institutions believed that patients did not require childcare services despite prior referrals and past financial support, highlighting the disparity between providers’ perceptions and the actual need for childcare services [24].

### Strategies for addressing childcare needs of cancer patients

#### The role of healthcare providers

The gap between patients’ childcare needs and providers’ perceptions can be bridged by collecting, analyzing, and interpreting relevant data on the childcare needs of eligible patients (see Table 1). Integrating childcare needs assessments into general patient evaluations or as part of patients’ healthcare information collection will provide actionable data for future interventions. Ideally, this should focus on understanding the parental status of patients, number of children, utilization of childcare services, and identifying unmet needs for childcare. The information obtained can be used to formulate hospital policies that promote childcare access, including the direct provision of childcare services and patient education on accessing these services. Needs assessments should extend beyond pre-assessment to post-intervention appraisals, with an aim to redesign or modify strategies as needed. To be effective, a needs assessment for childcare support should be approached from the patient’s perspective to prevent patient-provider discordance and ensure that patients’ needs are understood and duly represented [65].

**Table 1:** Strategies for addressing the childcare needs of cancer patients

Intervention	Examples
**Provider-led interventions**	Needs assessment Patient advocacy Data collection Patient education Screening with NCCN distress thermometer
**Hospital-based interventions**	Financial support schemes Non-traditional treatment schedules Patient counseling and education Raising provider awareness of childcare needs Facility-based childcare centers
**Community/social systems**	Non-traditional hour childcare Family and friends’ networks Home-based child-care centers Paid nannies
**Government-funded programs**	Child and Dependent Care Tax Credits (CDCTC) Childcare and Development Fund (CCDF) Childcare and Development Block Grants (CCDBG) Temporary Assistance for Needy Families (TANF)
**Professional organizations**	Raising physician awareness Information dissemination Grant allocation Stakeholder engagement Grassroots advocacy Providing patient resources Policy statements
**Insurance-funded caregiving support programs**	Payment for family caregivers

Furthermore, the National Comprehensive Cancer Network’s (NCCN) treatment guidelines include a patient distress section that identifies childcare as a stressor that could negatively impact a patient’s ability to manage a cancer diagnosis [66]. Therefore, screening for stressors using the NCCN distress thermometer is recommended for cancer patients when appropriate. Incorporating the distress thermometer into patient screening by healthcare providers will simplify the process of identifying patients with childcare needs for prompt intervention or assistance.

#### Hospital-level interventions

Certain cancer care institutions have identified the crucial role hospital-based childcare services could play in improving cancer treatment outcomes [25]. A survey of oncology providers demonstrates a prevailing sentiment that the absence of center-based childcare services is a source of stress for patients, and their availability could increase treatment efficiency through improved treatment adherence and fewer missed appointments [9, 67]. Currently, only one National Cancer Institute (NCI) designated cancer treatment center in the U.S. offers childcare support services to patients [25]. Given patients’ preference for hospital-based childcare services [11], efforts to mitigate missed appointments or treatment interruptions through in-hospital childcare spaces for cancer patients will likely be successful. Other potential hospital-centered approaches that have been considered include financial support from hospital systems. A feasibility study conducted by Brown University to assess the effect of financial assistance for childcare on treatment adherence was primarily associated with improved treatment adherence and a decline in missed patient visits [25]. Further evaluation will be required to determine whether these programs will work on a larger scale and how they will influence patient childcare support.

In addition, integrating non-traditional chemotherapy infusion and clinic follow-up visit schedules into existing treatment structures and offering telehealth visits when feasible could minimize the limitations imposed by lack of childcare access on treatment compliance. Patient visits across the spectrum follow the conventional standards, generally occurring during working hours on weekdays across cancer treatment centers. There is currently no data on non-traditional treatment schedules. Still, we posit that an alternative schedule will afford cancer patients who are also parents to better coordinate and manage childcare demands and could potentially improve treatment adherence.

#### Community and social systems

Nontraditional-hour (NTH) childcare programs were initially designed to accommodate parents’ childcare needs outside standard working hours, including weekends [57, 68]. Due to restrictions to standard work hours and workdays, routine center-based childcare centers are ill-equipped to meet off-hour childcare support needs. Therefore, NTH services are crucial for cancer patients who require services outside of routine hours due to medical emergencies, treatment fatigue, and other complications that make it challenging to mind their children. The need for NTH childcare is not restricted to cancer patients alone; up to 40% of families across the U.S. require NTH childcare services [57]. Racial minorities and families below the poverty line make up the population with the greatest need for NTH services [57, 68]. Although the demand for NTH was assessed based on parental employment, and no study has been conducted to determine the specific needs of cancer patients, we believe that cancer patients seeking childcare for their children likely represent a group for whom NTH services will be highly beneficial.

Furthermore, monetary compensation for childcare through Family Friends and Neighbor care (FFN), care provided by close social networks, and financial assistance for licensed home-based non-family providers may also alleviate the childcare burdens for cancer patients. For example, a non-profit, home-based, volunteer-facilitated initiative called Nanny Angel Network was launched in Toronto, Canada, to provide free childcare support services to mothers receiving cancer treatment. The goal was to promote treatment adherence, address missed appointments, and improve the overall well-being of parents as they navigate the cancer journey [69]. Eighty-seven percent of patients who participated in the program showed improved adherence to treatment schedules [34]. In a separate study, patients who received financial assistance toward childcare support during cancer treatment had no missed treatment or follow-up appointments [22].

#### Governmental funded programs

Another potential strategy is utilizing childcare-directed state and federal assistance programs. Childcare subsidies and child tax credits are government-funded programs primarily instituted to spur labor force participation and subsidize childcare for working parents [70, 71]. Through these programs, families can claim up to $6000 in Child and Dependent Care Tax Credit (CDCTC) for two dependents annually or $2000 per dependent [72]. CDCTC maximizes the benefits for working families by encouraging dual-parent families with one or two working parents. It also motivates welfare dependents to transition to employment and self-reliance [71, 73, 74]. However, cancer patients with dependents who are also likely to be unemployed may derive minimal to no benefit from income-based childcare support programs and may have to depend on other funding sources [75].

The Child Care and Development Block Grant (CCDBG) is another federal funding program established to provide high-quality childcare access for families in the lower socio-economic strata. One could argue that many cancer patients fall into this category and should, therefore, be recognized as a subset of families for whom special consideration should be extended to increase access to funding. Funding through other forms of grants like the Childcare and Development Fund (CCDF) [74], which provides funding to low-income families with children under 12 years, and Temporary Assistance for Needy Families (TANF) could also reduce out-of-pocket costs. Patients in the lower socio-economic strata may have limited knowledge of how best to access government-sourced subsidies and tax credits. Hospitals and care providers can bridge this knowledge gap through the provision of resources for accessing funding and unpaid tax filing assistance to patients who may require these services. This strategy is already being used by safety net hospitals in New York and should be expanded to include cancer patients with childcare needs [76].

#### Professional organizations

Professional medical organizations have a responsibility to support physicians in delivering high-quality patient care and to advocate for both physicians and their patients. Medical oncology societies, such as the American Society of Clinical Oncology (ASCO), which has over 40,000 members from 150 countries, tend to have a broad reach. These professional oncologic bodies can utilize their extensive resources, including governmental structures, industrial connections, and diverse expertise, to improve childcare access for oncology patients. By leveraging their size and global influence, cancer societies can be instrumental in raising awareness, educating members about the existing childcare gaps for cancer patients, engaging external stakeholders, and promoting grassroots advocacy. Other meaningful interventions include creating workgroups to develop resources for patient education and access optimization, establishing policy positions that promote childcare access, allocating grants to encourage further research, and establishing childcare intervention projects.

#### Insurance-funded caregiving support programs

Millions of family members serve as caregivers for children or ailing elderly relatives. These caregiving responsibilities can be financially draining, as family members often need to balance work outside the home with caregiving duties. In recognition of the economic burden of caregiving, many states across the U.S. have established payment programs to support family caregivers. For example, the Consumer Directed Personal Assistance Program (CDPAP) was launched by New York Medicaid to support family members who provide home care for ailing Medicaid recipients [77]. Through this program, family members can enroll as caregivers and receive remuneration from Medicaid. Eligible caregivers include the children of the recipient, close relatives, and friends, although legal spouses are exempt. CDPAP aims to improve access to home care services for seniors and disabled patients through the program.

Currently, no similar assistance programs exist for cancer patients with young children. Extending such programs to fund childcare services for cancer patients could help address this population’s significant unmet childcare needs.

## Conclusion

The rising cancer incidence among patients in the reproductive age group with dependent children has created a growing need for childcare support in this population. However, childcare services remain unavailable for most parents undergoing cancer treatment. Providing NTH childcare services, home-based or institution-based childcare services, and financial assistance through government-funded programs could bridge the current gap in childcare needs for parents dealing with a cancer diagnosis.

## Data Availability

No datasets were generated or analysed during the current study.
